# Factors influencing the allocation of China’s development assistance for health

**DOI:** 10.7189/jogh.08.020502

**Published:** 2018-12

**Authors:** Yingxi Zhao, Kaci Kennedy, Kun Tang

**Affiliations:** 1 University of Washington, Department of Global Health, Seattle, Washington, USA; 2 Peking University Institute for Medical Humanities, Beijing, China; 3 Tsinghua University School of Public Policy and Management, Beijing, China; 4 Peking University Department of Global Health, Beijing, China

## Abstract

**Background:**

China has been described as a “rogue” donor suspected of using foreign assistance to obtain raw materials, promote exports, and strengthen its business links with aid recipient countries. However, the factors influencing China’s development assistance policy have rarely been analysed, particularly those related to its health aid. This study explores the factors that could affect the allocation of China’s development assistance for health (DAH) from three key aspects: recipient need, recipient merit, and donor interest.

**Methods:**

Analysis was based on cross-sectional data of China’s DAH from 2006 to 2014. This study explores the following characteristics of the 82 recipient countries identified in the data: the association between China’s allocation of DAH and recipient need (measured by Gross Domestics Product [GDP] per capita and all-cause Disability-Adjusted Life Years [DALYs]), recipient merit (measured by government effectiveness and human rights violations) and donor interests (measured by China’s export, United Nations voting alignment, and recipient natural resources). A stratified analysis was conducted to understand these associations in different development contexts and the factors that influenced each type of DAH.

**Results:**

Multivariate Spearman correlation suggested that the most significant factors influencing China’s allocation of DAH were the recipient countries’ GDP per capita (*r* = -0.31941, *P* = 0.0049) and human rights conditions (*r* = -0.23227, *P* = 0.0435). Health workforce was associated with medical team deployment (*r* = -0.20929, *P* = 0.0715), while malaria DALYs was associated with anti-malaria center establishment (*r* = 0.46473, *P* < 0.0001). According to the sub-group analysis, donor interests such as trade and natural resources only slightly influenced DAH allocation.

**Conclusion:**

Recipient need and merit strongly influence China’s DAH allocation while donor interests only slightly influence DAH allocation in certain development contexts.

The official development assistance (ODA) landscape has changed markedly in recent years; the purpose, quantity, and modalities have evolved along with the identities of aid donors and recipients. In 2015, net ODA from members of the Development Assistance Committee (DAC) of the Organization for Economic Co-operation and Development (OECD) reached 146.68 billion US$, the highest real level of ODA recorded [1]. Since 2000, ODA from OECD-DAC members has increased from 53.9 billion US$, an increase of almost 200 percent [1]. Non-OECD-DAC donors have also significantly increased aid levels. ‘South-South cooperation’ (SSC), the development cooperation model between the Global South, has become much more prominent over the last decade: estimates of total South-South cooperation stand at about 15.3 billion US$ in 2008 (in current prices), or 9.5 percent of total development cooperation [2]. The rapid economic growth of many major developing countries has led to their greater role in international affairs.

Past studies have concluded that ODA allocation rationale is not limited to developing countries’ needs but also includes donor self-interest. A recipient country's income level and humanitarian needs are certainly factors shaping the allocation of DAC aid [3]. There are also conditions attached to aid: donor countries may request the recipient country to vote in line with it in the international fora such as the United Nations [4]. Moreover, recipient countries may be requested to pursue particular economic or financial policies or establish governance structures that donors deem necessary for their aid to be effective in promoting growth and reducing poverty, thus recipient countries’ own system is also an important factor influencing ODA allocation [4]. Empirical analysis revealed three broad categories of factors that may influence ODA allocation [5,6].

(1) Donor Interests: the interests of donor countries when deploying aid, including benefits in trade and commerce, foreign policy, etc.

(2) Recipient Need: the level of development assistance need of a recipient country, including overarching development needs such as poverty reduction and peace-building as well as aid-specific needs such as disease burden reduction.

(3) Recipient Merit: the quality of policies and institutions in a recipient country, given that a recipient's economic policies and system of government may affect donor aid allocation.

China has a long history of deploying foreign aid as an indispensable component of its foreign diplomacy. Since 1950, the Chinese government has provided various forms of foreign aid to 160 countries and over 30 international organizations [7]. China's use of development assistance for health (DAH) as a geopolitical tool dates back to its engagement with neighboring Southeast Asian countries in the 1950s. Its engagement expanded beyond Asia into African countries in 1963 when it dispatched its first medical team to Algeria. [8,9]. According to China’s 2014 foreign aid white paper, health (alongside other social development sectors) is one of the key focus areas of China’s ODA [10].

China's health-related development assistance has traditionally taken three forms: medical teams, infrastructure, and knowledge transfer [9]. China has dispatched medical teams as a traditional and cost-effective approach to providing health aid to recipient countries. In the past 50 years, China has dispatched over 24 000 medical workers to nearly 120 medical centers worldwide. In the last decade, China has placed heavy emphasis on health care infrastructure and medical supplies while continuing to deploy medical teams to the least developed countries. Since 2000, China has supported nearly 150 hospital construction projects in Africa and 180 batches of medical supplies – including drugs – to low- and middle-income countries facing humanitarian emergencies or natural disasters [9]. In addition to investing in medical teams and health infrastructure, China has also invested in transferring its domestic disease control knowledge to other developing countries through South-South cooperation. In addition to China’s 30 years of rapid economic growth, China has also made significant achievements in reducing maternal and infant mortality, gaining control of malaria, and providing universal coverage of child immunizations [11]. China has actively promoted the "Chinese experience" in infectious disease control; it has conducted trainings for disease prevention and treatment and provided in-kind antimalarial drugs, flu and cholera vaccines to other developing countries [9]. The 2006 Forum on China–Africa Cooperation (FOCAC) summit led to China’s anti-malaria campaign, and the first treatment center was established in Liberia. Since 2006, the Chinese government has provided US$ 100 million in direct investment to fund the establishment of 30 anti-malaria centers [12].

However, the intentions driving China’s foreign aid allocation strategies have often been questioned. China is suspected of obtaining raw materials, promoting exported products, and strengthening business linkages with recipient countries in exchange for aid [13]. Naím characterized China’s development aid as “rogue aid” [14]: claiming that its aid allocation is unrelated to the need of developing countries and driven by China’s own interests such as gaining greater access to resources and/or boosting international alliances. “Rogue aid” is believed to undermine the development efforts of traditional Western donors. Dreher and Fuchs’ analysis of China's aid allocation from 1956-2006 showed that although China's aid allocation is largely independent of a recipient country's endowment of natural resources, there is some relationship with the economic development status of a country and certain geopolitical factors (such as the diplomatic recognition of Taiwan or the alignment of voting behavior at the UN) [15].

China’s health-related assistance has not been without criticism. Health-specific aid has also been considered a Chinese commercial policy tool, as the Chinese state enterprises has been largely involved in the DAH process; domestic pharmaceutical firms have provided their products as “health aid”, which could potentially expand the market share of Chinese pharmaceutical products in settings such as Africa [16]. Often used to support this argument are quotes such as this issued by the Ministry of Health in 2003: “China’s health aid should not only serve China’s foreign policy, but also act as a broker for economic development in China and recipient countries” [17].

Few study have used a quantitative approach to either support or refute these claims of Chinese aid allocation, which may partly be due to limited data sources on China’s DAH. Existing literature suggests that there is no relationship between China’s DAH and a recipient country’s natural resources (oil rents, natural gas rents, coal rents, mineral rents, and forest rents) [18] but shows mixed results in terms of economic interests (petroleum imports, China’s foreign investment, and China’s imports and exports) [12,19]. In contrast, diplomatic recognition of Taiwan (the so-called “One-China” policy) and a recipient country’s economic situation (GDP per capita) are found associated with medical team deployment [15]. The previously referenced studies excluded important factors such as political interest and failed to adjust for the development status of a recipient country. Incorporating these elements could uncover the unbiased associations of social development, geopolitics and economic factors with China’s aid behavior.

This paper aims to analyse the associations of China’s DAH allocation with recipient need, recipient merits and donor interests to explore the factors that influence China’s DAH allocation. The results of this study are of vital importance to expand existing knowledge of China’s DAH allocation.

## METHODS

### Data design

Our model used cross-sectional data from 2006-2014. 137 ODA recipient countries were identified (the standard was per capita GNI<US$ 12275 in 2010, China was excluded) [20], and 45 countries that are not recipients of China’s DAH were excluded. 10 countries were further excluded due to incomplete data (Dominica, Kosovo, Marshall Islands, Moldova, Nauru, Democratic People’ s Republic of Korea, Palau, St. Kitts and Nevis, Tuvalu, Yugoslavia). The remaining 82 OECD ODA recipient countries who received China’s DAH were examined.

### Variables

The outcome variable in this study was China’s DAH level to all recipient countries before 2011. DAH level refers to the cumulative types of health aid, including “medical team deployment”, “hospital construction” and “anti-malaria center construction”. In this study, the DAH level index is defined as a three-scale indicator (1, 2, 3):

“1” refers to one kind of aid;
“2” refers to the combination of two types of aid;
“3” refers to the provision of all three types of aid.

AidData – the first systematic and only publicly available database on China’s development aid – is the source of this study’s data. Health-related project-level data was extracted from the database, and the DAH level index was calculated accordingly. Data was further validated by expert interviews and internal material from China National Health Development Research Center (a National Health and Family Planning Commission affiliated think tank).

Based on the information available from earlier studies, our allocation model includes three categories of explanatory variables: recipient need, recipient merit, and donor self-interest (**Table 1**).

**Table 1:** Summary of potential influencing factors of China’s DAH

Category	Influencing factors	Data source	Note
**1 Recipient need**	**1a** Recipient country GDP per capita	World Development Indicators (http://data.worldbank.org/)	Average of GDP per capita 2006-2014, in current US$
	**1b** Recipient country all-cause DALYs	IHME Global Burden of Disease (http://ghdx.ealthdata.org/ )	Data from 2010
	**1c** Recipient country health workforce	World Health Organization	Data from World Health Statistics 2011, physicians number per 10 000 population between 2000-2010
	**1d** Recipient country hospital beds	World Health Organization	Data from World Health Statistics 2011, hospital beds number per 10 000 population between 2000-2009
	**1e** Recipient country malaria DALYs	IHME Global Burden of Disease (http://ghdx.healthdata.org/)	Data from 2010
**2 Recipient merit**	**2a** Recipient country government effectiveness	Worldwide Governance Indicators (http://info.worldbank.org/governance/wgi/#home)	Data from 2011, a scale ranging from -2.5 to 2.5 with higher values corresponding to better performance
	**2b** Recipient country human rights violation	Political terror scale (http://www.politicalterrorscale.org/)	Data from 2011, a scale between 1 and 5, with higher numbers indicating more human rights abuses
**3 Donor Interest**	**3a** Trade level	UN Comtrade (https://comtrade.un.org/)	The proportion of China’s export to the recipient’s total import in 2011
	**3b** Voting alignment in the UN	United Nations General Assembly Voting Data (https://dataverse.harvard.edu/dataset.xhtml?persistentId=hdl:1902.1/12379)	Average across 2006-2014, ranged from –1 (least similar interests) to 1 (most similar interests)
	**3c** Recipient country natural resource rent	World Development Indicators	Average across 2006-2014, the total natural resource rents (as a % of GDP)

UN – United Nations, GDP – gross domestic product, IHME – Institute for Health Metrics and Evaluation

#### Recipient need

Five indicators were included: the former two indicators (1a and 1b) were used for general analysis and the latter three (1c, 1d, and 1e) were used in type-specific analysis.

(1a) Recipient Gross Domestic Product (GDP) per capita was retrieved from the World Development Indicators Database and was defined as the average GDP per capita from 2006 to 2014 in current US$.
(1b) Recipient age-standardized Disability Adjusted Life Years (DALYs) is an indicator commonly used to quantify the burden of disease from mortality and morbidity. Data was obtained from the IHME Global Burden of Disease 2010 study [21].
(1c) Recipient country health workforce was retrieved from the World Health Statistics [22], which was defined as the recipient country’s physician number per 10 000 population, from 2000-2010.
(1d) Recipient country hospital beds was retrieved from the World Health Statistics, which was defined as the recipient country’s hospital beds number per 10 000 population, from 2000-2009.
(1e) Recipient countries’ malaria DALYs, which was the malaria-specific burden of disease retrieved from the IHME study [21].

#### Recipient merit

(2a) Recipient country government effectiveness data was measured using index developed by Kaufmann [23]. The index ranged from -2.5 to 2.5, with higher values corresponding to better performance.
(2b) Human rights violations data were retrieved from the Political Terror Scale’s data in 2011. The index is measured on a scale of 1 to 5, with higher numbers indicating more human rights abuses [5].

#### Donor need

(3a) Trade level was defined as the flow of China’s export to a recipient, as a percentage of recipient’s overall imports in 2011, using data from UN Comtrade.
(3b) Voting alignment in the UN, which is a commonly used indicator was attained by China and the recipient countries’ voting affinity at the UN General Assembly from 2006 to 2014, value ranged from –1 (least similar interests) to 1 (most similar interests). The detailed methodology of this indicator could be found in Signorino and Ritter’s study [24].
(3c) Recipient country natural resource was defined as the sum of oil rents, natural gas rents, coal rents (hard and soft), mineral rents, and forest rents, as a percentage of recipient GDP from 2006 to 2014. Data was retrieved from the World Development Indicators Database.

### Data analysis

Univariate analyses were conducted using the Spearman rank correlation to understand the association between each influencing factor and DAH level. Spearman correlation was further conducted to understand whether recipient GDP per capita, recipient DALYs, recipient government effectiveness, recipient human rights violations, trade level, voting alignment at UN, and/or recipient natural resource rent influence China’s allocation of DAH (the DAH level), while adjusting for each other.

To evaluate the modifying effects of recipient development status, stratified analyses were performed under each development stage. Based on OECD classification, countries were categorized according to per capita GNI into the following three groups: least developed countries (n = 41), low-income countries and lower middle-income countries and territories (n = 21, per capita GNI<US$ 3975 in 2010), and upper middle-income countries and territories (n = 20, per capita GNI US$ 3976 ~ 12 275 in 2010) [20]. Spearman correlation coefficients were reported within each category of development status to understand whether the three categories of explanatory variables influence China’s allocation of DAH while adjusting for each other. Furthermore, each type of DAH (ie, medical team deployment, hospital construction, and anti-malaria centers) were adjusted for additional factors: medical team analysis (recipient country DALYs and health workforce), hospital analysis (recipient country DALYs and hospital beds), and anti-malaria center analysis (recipient country malaria DALYs). For the anti-malaria center analysis, all-cause DALYs was not included due to collinearity.

All statistical analyses were performed using SAS Version 9.3 (SAS Institute Inc., Cary, North Carolina, USA), and significant test results were reported at *P* < 0.05 and *P* < 0.1 levels.

## RESULTS

**Figure 1:**
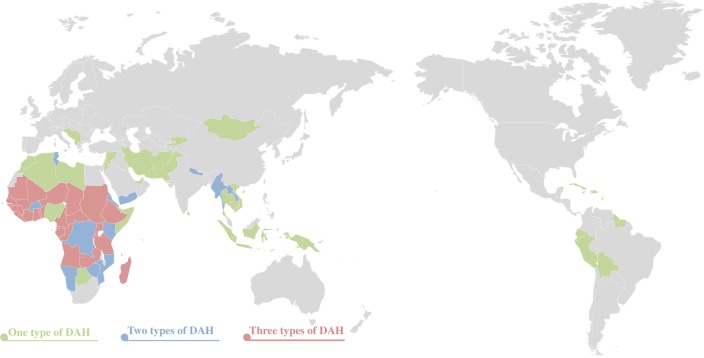
Recipients of China’s development assistance for health and DAH level (until 2011).

Between 1963 and 2011, a total of 82 countries were identified as recipient countries of China’s development assistance for health; among them, 41 countries were least developed countries (LDCs) according to the OECD definition. Of the 82 recipient countries, 26 received all three types of health aid (medical team deployment, hospital construction and anti-malaria center construction), 20 countries received two types of aid, and 36 countries received one type of health aid (**Figure 1**).

**Table 2** shows the results of the univariate and multivariate analysis of the association between each influencing factor and China’s health aid level. Recipient country GDP per capita was significant both in univariate (*r* = -0.46093, *P* < 0.0001) and multivariate analysis (*r* = -0.31941, *P* = 0.0049). Recipient country all-cause DALYs (*r* = 0.20155, *P* = 0.0694), government effectiveness (*r* = -0.39653, *P* = 0.0002), and natural resource rent (*r* = 0.27428, *P* = 0.0126) were significantly associated with health aid level in the univariate analysis, but were insignificant in the multivariate analysis (*r* = 0.15294, *P* = 0.1872; *r* = -0.19208, *P* = 0.0965; and *r* = 0.12583, *P* = 0.2788 respectively). Recipient country human rights violations was insignificant in the univariate analysis (*r* = 0.02184, *P* = 0.8456) but was significant in the multivariate analysis (*r* = -0.23227, *P* = 0.0435).

**Table 2:** Spearman correlation coefficients between DAH level and influencing factors: results of the univariate and multivariate analyses

Influencing factors	Univariate analysis		Multivariate analysis	
	**Coefficients**	***P*-value**	**Coefficients**	***P*-value**
**1a** Recipient country GDP per capita	-0.46093	<0.0001†	-0.31941	0.0049†
**1b** Recipient country all-cause DALYs	0.20155	0.0694*	0.15294	0.1872
**2a** Recipient country government effectiveness	-0.39653	0.0002†	-0.19208	0.0965*
**2b** Recipient country human right violation	0.02184	0.8456	-0.23227	0.0435†
**3a** Trade level	0.09680	0.3869	-0.01130	0.9228
**3b** Voting alignment in the UN	-0.09417	0.4001	-0.10002	0.3900
**3c** Natural resource rent	0.27428	0.0126†	0.12583	0.2788

GDP – gross domestic product, UN – United Nations, DALY – disability-adjusted life year

*P < 0.1.

†*P* < 0.05.

Stratified analysis was conducted to fully evaluate the modifying effect of recipient development status on China’s allocation of its development assistance for health (**Table 3**). Among the least developed countries, DAH level was associated with natural resource rent. Among other lower income and lower middle-income countries, health aid level was independent of all influencing factors. Among upper middle-income countries, the trade level between China and recipient countries was associated with the level of DAH provided, with a coefficient of -0.53412 and *P* level of 0.0491, indicating that a lower trade level was significantly associated with more health aid.

**Table 3:** Spearman correlation coefficients between DAH level and influencing factors: stratified by development status

Influencing factors	Least developed countries (n = 41)		Lower income and Lower middle income countries (n = 21)		Upper middle income countries (n = 20)	
	**Coefficients**	***P*-value**	**Coefficients**	***P-*value**	**Coefficients**	***P*-value**
**1a** Recipient country GDP per capita	-0.02792	0.8735	-0.22486	0.4204	0.10356	0.7246
**1b** Recipient country all-cause DALYs	0.21920	0.2058	0.05563	0.8439	0.35880	0.2077
**2a** Recipient country government effectiveness	-0.11776	0.5005	0.05409	0.8482	-0.04936	0.8669
**2b** Recipient country human right violation	-0.21487	0.2151	-0.11554	0.6818	-0.12654	0.6664
**3a** Trade level	0.06313	0.7181	-0.05348	0.8499	-0.53412	0.0491†
**3b** Voting alignment in the UN	-0.16066	0.3566	-0.02198	0.9380	-0.23426	0.4202
**3c** Natural resource rent	0.29892	0.0811*	-0.03012	0.9152	0.31022	0.2804

DAH - development assistance for health, GDP – gross domestic product, UN – United Nations, DALY – disability-adjusted life year

*P < 0.1.

†*P* < 0.05.

Finally, the associations between various factors influencing China’s DAH and each health aid type was analyzed; **Table 4** shows the result of the multivariate analysis. Whether China sent a medical team to a recipient country was associated with the recipient country’s health workforce density (*r* = -0.20929, *P* = 0.0715). In contrast, China’s hospital construction was independent of all influencing factors, including the density of hospital beds of the recipient country (79 countries were included in this sub-group analysis due to missing value of three countries). As for China’s anti-malaria centers, the recipient country’s malaria DALYs significantly influenced China’s DAH allocation (*r* = 0.46473, *P* < 0.0001). Human rights violations were also negatively associated with the establishment of a China’s anti-malaria center (*r* = -0.19235, *P* = 0.0960).

**Table 4:** Spearman correlation coefficients between different types of China’s DAH and influencing factors

Influencing factors	Medical team*		Hospital construction*		Anti-malaria center	
	**Coefficients**	***P*-value**	**Coefficients**	***P*-value**	**Coefficients**	***P-*value**
**1a** Recipient country GDP per capita	-0.06087	0.6039	-0.03537	0.7680	-0.04147	0.7221
**1c** Recipient country health workforce	-0.20929	0.0715*	–	–	–	–
**1d** Recipient country hospital beds	–	–	-0.07326	0.5408	–	–
**1e** Recipient country malaria DALYs	–	–	–	–	0.46473	<0.0001‡
**2a** Recipient country government effectiveness	-0.09735	0.4060	-0.13813	0.2472	-0.13393	0.2487
**2b** Recipient country human rights violation	-0.16843	0.1486	0.03929	0.7431	-0.19235	0.0960**†**
**3a** Trade level	0.11007	0.3472	-0.05387	0.6531	-0.05322	0.6480
**3b** Voting alignment in the UN	0.14313	0.2206	-0.06558	0.5841	-0.16715	0.1490
**3c** Natural resource rent	0.06214	0.5964	-0.02098	0.8612	0.10031	0.3386

*Additionally adjusted for all-cause DALYs.

**†***P* < 0.1.

‡*P* < 0.05.

## DISCUSSION

The present study is one of the few studies that analyzed China’s allocation of DAH using a quantitative approach. This study indicates that China allocated more health aid to countries with stronger development needs and better merit in terms of good governance and human rights records. We found no evidence that China’s DAH was directly associated with bilateral trade or UN voting affinity. There was a weak association between China’s DAH and natural resource rent, which diminished after adjusting for other confounding factors. Although China has been suspected of using foreign assistance to obtain raw materials, promote domestic exportations and strengthen its business ties with recipient countries, such suspicion appears to be invalid at least in the health sector.

### Strengths and limitations

This paper offers several contributions to existing knowledge on China’s DAH. First, this study collected the best available information on China’s aid channel and volume. Second, despite the similarity between health and other social development sectors like agriculture and education, DAH has largely been neglected by researchers of development aid. This paper provides an empirical analysis of factors determining the allocation of health aid, and explains the political and economic influence of DAH.

There are several potential limitations of the present study. First, the levels of China’s DAH was measured as a three-scale indicator rather than the actual volume of aid allocated. This was largely due to insufficient data, and the uncertainty in the accuracy of total aid volume provided in AidData, where we identified 40% of health projects had missing values. On one hand, using DAH level instead of total volume might not appropriately reflect the extent of China’s development assistance given that concessional loans and other development assistance for health (eg, emergency humanitarian aid) were not included. On the other, arbitrarily using categorical average to fill out missing value may lead to bias. In general, considering that there was no official data of China’s overseas development aid, these surrogate DAH level indicators were the best-available evidence for the present analyses. Second, also due to limited data, the author only used cross-sectional analyses, which cannot provide a cauxal relation of the influencing factors and China’s allocation of DAH.

### Recipient need

This study found that a recipient country’s GDP per capita and health status may influence China’s DAH allocation, as both recipient GDP per capita and DALYs were significantly associated with DAH allocation. It was found that significantly more health aid was allocated to least developed countries. In addition, evidence suggested that medical teams were sent to countries with poor health human resources and anti-malaria centers were established in countries with a high malaria burden. This shows that China’s health aid allocation was, to a certain extent, guided by recipient needs, which aligns with China’s recipient-driven mechanism indicated in the “Five Principles of Peaceful Coexistence” [25]. Meanwhile, in our analysis, half of all China’s DAH recipient countries are the least developed countries (LDCs). Although China did not have documented goals or strategies for health aid, it is important to note that China’s health aid complied to the Paris Declaration on Aid Effectiveness by prioritizing recipient countries’ preferences and health demands, , especially for medical teams and anti-malaria centers.

### Recipient merit

Government effectiveness was negatively associated with DAH level, and human rights violations were also a significant influencing factor of DAH allocation; sub-group analysis indicated that human rights violations were also associated with anti-malaria center allocation. It should be emphasized that in the political terror scale data set—which we use as a measure of human rights – higher positive values are associated with greater abuse of political rights. The negative coefficient for human rights violation with DAH level indicates that China’s health aid concentrates in political environments with a greater respect for human rights.

Recipient merit such as government policies and human rights records are important factors for many traditional donors. Countries such as France, Germany, and Japan all take human rights abuses into account when allocating foreign aid. Another example is the UK, which rewards good economic policies and democracies [5]. Some donors may even request recipient countries to improve government transparency or reduce human rights violations in order to increase aid effectiveness [4].

Different from western donors, China’s DAH reflects China’s “non-interference” principle - “not imposing any political conditions nor interfering in the affairs of the recipient countries” [5]. This approach has its own pros and cons: on one hand, it allows for recipient ownership and is responsive to a recipient government’s needs [26]; On the other hand, this may lead to a common concern such as corruption and aid effectiveness. Since most of China’s aid is tied - especially its hospital construction projects and details of the contracts and procurement are scarce, this could easily to skepticism. However, the cross-sectional data could not lead to the casual relation on this issue.

### Donor interests

Our study found that bilateral trade was associated with DAH allocation only in upper middle-income countries, while the negative coefficient suggested that more health aid flew to countries with weaker trade relations with China. It has always been hypothesized that China has used DAH as a brokerage to promote bilateral trade [16]. It’s worth noticing the Chinese government’s encouragement of its agencies and commercial entities to “closely mix and combine foreign aid, direct investment, service contracts, labor cooperation, foreign trade and export” [27] to maximize feasibility and flexibility of the projects to meet local realities in a recipient country. Therefore, many of China’s DAH programs—particularly hospital construction—were implemented under a larger development aid package including projects such as railways, highways, port construction, and civilian transportation [9]. Consistent with China’s “mutual benefit and win-win” principle, far-reaching aid packages could potentially be a driver of China’s export to the recipient countries [10]. However, the association was only found in upper middle income countries but not in least developed countries, which also may reflect the humanitarian characteristics of China’s DAH.

For political considerations, past research has shown strong evidence that politics plays an important role in the allocation of aid money and medical staff to recipient countries in all phases of China’s aid program [15]. However, our analysis suggested that UNGA voting affinity was not associated with overall DAH allocation. This may suggest that China adhered to its principles of “not imposing any political conditions nor interfering in the affairs of the recipient countries” [10]. However, it was hard to conclude that China’s health aid had absolutely no political considerations as Taiwan recognition has still been one of the key influencing factors that determines China’s DAH - and overall - aid allocation. For example, China suspended sending medical teams to Liberia (in 1989), Central African Republic (in 1991) and Niger (in 1992) due to the recipient countries’ foreign relations with Taiwan. The medical teams resumed operations after the recipient countries suspended diplomatic relations with Taiwan in 2005, 1996 and 1998 respectively [28]. In brief, UNGA voting affinity is not associated with DAH level, while other political issue might potentially influence the allocation of DAH.

Our study found that a recipient country’s natural resources were not associated with DAH allocation after adjusting for other confounders; this finding is consistent with the analysis of Grépin et al [18]. Additionally, our study also found that natural resource rent was positively associated with DAH level in LDCs. China frequently provided DAH and low-interest loans to countries that relied on commodities such as oil and mineral resources. In such cases, the recipient country usually suffered from low credit ratings and encountered great challenges obtaining funding from the international financial market. Alternatively, China makes financing relatively easily accessible, albeit with certain conditions (for example Taiwan recognition) [29]. Nonetheless, our study found that a recipient country’s natural resources are not an influencing factor for China’s DAH allocation.

China is neither a signatory member of the Paris Declaration on Aid Effectiveness nor committed to the Busan Partnership for Effective Development Cooperation on a “voluntary basis” [30], which indicates China’s position as an “outlier” of development aid. The past decade has witnessed China’s increasing ambition to play a more active role in global governance especially in terms of promoting the South-south cooperation model that challenges the set of standards long-built by traditional donors. China has established and committed a sizeable package of new development aid and cooperation with African and Asian countries, including but not limited to the launch of the Belt and Road initiative, establishment of the Asian Infrastructure Investment Bank (AIIB) and the New Development Bank (NDB) [31]. Further observation of China’s move in global health and development is needed to understand China’s DAH behaviour.

## CONCLUSIONS

By exploring the potential factors that could affect the allocation of China’s development assistance for health, this study shows that China allocated more health aid to countries with stronger development needs and stronger recipient merit in terms of good governance and human rights records. We found no direct evidence that China’s DAH was directly associated with bilateral trade or UN voting affinity.

## References

[1] OECD. Official Development Assistance 2014. Available: http://www.oecd.org/statistics/datalab/oda2012.htm. Accessed: 28 March 2017.

[2] UNDESA. International Development Cooperation Report 2010. Available: http://www.un.org/en/ecosoc/newfunct/pdf/10-45690(e)(desa)development_cooperation_for_the_mdgs_maximizing_results.pdf. Accessed: 28 March 2017.

[3] Nunnenkamp P, Thiele R (2006). Targeting aid to the needy and
deserving: Nothing but promises?. World
Econ.

[4] Dreher A, Nunnenkamp P, Thiele R (2011). Are ‘new’ donors different?
comparing the allocation of bilateral aid between nonDAC and DAC donor
countries.. World
Dev.

[5] Hoeffler A, Outram V (2011). Need, merit, or
self-interest—what determines the allocation of
aid?. Rev Dev
Econ.

[6] Nunnenkamp P, Öhler H (2011). Aid allocation through various official
and private channels: Need, merit, and self-interest as motives of German
donor.. World
Dev.

[7] Information Office of the State Council. China’s Foreign Aid White Paper. 2011. Available: http://english.gov.cn/archive/white_paper/2014/09/09/content_281474986284620.htm Accessed: 25 June 2018.

[8] Boynton XL. China's Emerging Global Health and Foreign Aid Engagement in Africa. Center for Strategic & International Studies, 2011.

[9] Li RJ. China Development Report on South-South Cooperation. Beijing: China Intercontinental Press; 2015.

[10] Information Office of the State Council. China’s Foreign Aid White Paper. 2014. Available: http://english.gov.cn/archive/white_paper/2014/08/23/content_281474982986592.htm Accessed: 25 June 2018.

[11] Lee L (2004). The current state of public health in
China.. Annu Rev Public
Health.

[12] Liu P, Guo Y, Qian X, Tang S, Li Z, Chen L (2014). China’s distinctive engagement in
global
health.. Lancet.

[13] Pehnelt G. The political economy of China's Aid Policy in Africa. Jena Economic Research Paper. 2007;2007:051.

[14] Naím M. Rogue Aid. Foreign Policy. 2007.Available at: https://foreignpolicy.com/2009/10/15/rogue-aid/. Accessed: 1 July 2018.

[15] Dreher A, Fuchs A (2015). Rogue aid? An empirical analysis of
China’s aid allocation.. Can J
Econ.

[16] Huang Y (2014). Domestic politics and China’s
health aid to Africa.. China An International
Journal..

[17] Huang X. Health aid work is planning for a new level. CPPCC News. 2003. Available: http://gb.oversea.cnki.net/kcms/detail/detail.aspx?QueryID=17&CurRec=6&DbCode=CCND&filename=RMZX20031210ZZZ3&dbname=CCNDHIS Accessed: 25 June 2018.

[18] Grepin KA, Fan VY, Shen GC, Chen L (2014). China’s role as a global health
donor in Africa: what can we learn from studying under reported resource
flows?. Global
Health.

[19] Shajalal M, Xu J, Jing J, King M, Zhang J, Wang P (2017). China’s engagement with
development assistance for health in Africa.. Glob
Health Res
Policy..

[20] OECD. DAC List of ODA Recipients effective for reporting on 2011 flows. Available: http://www.oecd.org/dac/stats/documentupload/DAC%20List%20used%20for%202011%20flows.pdf. Accessed: 30 March 302017.

[21] Murray CJ, Ezzati M, Flaxman AD, Lim S, Lozano R, Michaud C (2012). GBD 2010: design, definitions,
and
metrics.. Lancet.

[22] World Health Organization. World Health Statistics 2011. Available: http://www.who.int/whosis/whostat/2011/en/. Accessed: 30 March 2017.

[23] Kaufmann D, Kraay A, Mastruzzi M. Governance Matters VIII. Governance Indicators for 1996–2008. Available: http://documents.worldbank.org/curated/en/598851468149673121/pdf/WPS4978.pdf. Accessed 25 June 2018.

[24] Signorino CS, Ritter JM (1999). Tau-b or Not Tau-b: Measuring the
similarity of foreign policy positions.. Int Stud
Q.

[25] Yang H, Acharya SP, Liu P, Guo Y (2014). Development assistance for health given
to Nepal by China and India: a comparative
study].. Global
Health.

[26] Brant P. Chinese aid and the aid effectiveness agenda. Available: http://www.thebrokeronline.eu/layout/set/print/Blogs/Busan-High-Level-Forum/Chinese-aid-and-the-Aid-Effectiveness-Agenda. Accessed: 29 June 2017.

[27] Piao YJ (2006). The evolution and future trend of
China’s direct investment in Africa.. Overseas
Investment & Export
Credits..

[28] Li AS (2009). History, scale and impact of
China’s medical team.. Foreign Affairs
Review..

[29] Sun Y. China’s aid to Africa: Monster or Messiah? Available: https://www.brookings.edu/opinions/chinas-aid-to-africa-monster-or-messiah/. Accessed: 30 April 2017.

[30] Council on Foreign Relations. Busan High-Level Forum: From Dead Aid to Better Development? Available: https://www.cfr.org/report/busan-high-level-forum-dead-aid-better-development. Accessed: 26 June 2017.

[31] Tang K, Li Z, Li W (2017). China’s Silk Road and global
health.. Lancet.

